# Development of a Fluorescence Resonance Energy Transfer (FRET)-Based DNA Biosensor for Detection of Synthetic Oligonucleotide of *Ganoderma boninense*

**DOI:** 10.3390/bios3040419

**Published:** 2013-12-12

**Authors:** Noremylia Mohd Bakhori, Nor Azah Yusof, Abdul Halim Abdullah, Mohd Zobir Hussein

**Affiliations:** 1 Chemistry Department, Faculty Science, Universiti Putra Malaysia, Serdang, Selangor 43400, Malaysia; E-Mails: noremyliamb@gmail.com (N.M.B.); halim@science.upm.edu.my (A.H.A.); mzobir@science.upm.edu.my (M.Z.H.); 2 Institute of Advanced Technology, Universiti Putra Malaysia, Serdang, Selangor 43400, Malaysia

**Keywords:** *Ganoderma boninense*, DNA biosensor, quantum dot, fluorescence resonance energy transfer

## Abstract

An optical DNA biosensor based on fluorescence resonance energy transfer (FRET) utilizing synthesized quantum dot (QD) has been developed for the detection of specific-sequence of DNA for *Ganoderma boninense*, an oil palm pathogen. Modified QD that contained carboxylic groups was conjugated with a single-stranded DNA probe (ssDNA) via amide-linkage. Hybridization of the target DNA with conjugated QD-ssDNA and reporter probe labeled with Cy5 allows for the detection of related synthetic DNA sequence of *Ganoderma boninense* gene based on FRET signals. Detection of FRET emission before and after hybridization was confirmed through the capability of the system to produce FRET at 680 nm for hybridized sandwich with complementary target DNA. No FRET emission was observed for non-complementary system. Hybridization time, temperature and effect of different concentration of target DNA were studied in order to optimize the developed system. The developed biosensor has shown high sensitivity with detection limit of 3.55 × 10^−9^ M. TEM results show that the particle size of QD varies in the range between 5 to 8 nm after ligand modification and conjugation with ssDNA. This approach is capable of providing a simple, rapid and sensitive method for detection of related synthetic DNA sequence of *Ganoderma boninense*.

## 1. Introduction

Oil palm has become an important source for vegetable oil in Asia, especially for Malaysia and Indonesia. This valuable resource is facing a serious problem on infection called basal stem rot (BSR) caused by a species known as *Ganoderma boninense* [1]. If not detected at early stage, the plants will end up dying; no known treatment can save the plants once the symptoms appear. BSR causes the appearance of fruiting bodies at the base of the stem, unopened spears, yellowing crown and deep cracks at the stem’s base [2].

Recently, studies on nanoparticles as sensor for diagnosis and detection of diseases has become of great interest among researchers. Quantum dot (QD) has been reported to have capabilities to overcome the limitations of organic dyes. QD is a semiconductor (CdSe, CdS, CdTe) that has diameter range between 2 to 10 nm which has advantages in optical applications. QD has unique properties such as broad absorption and narrow emission bandwidth that is ideal for simultaneous detection of multiple fluorophores [3]. In addition, QD is resistant to photobleaching, size-tunable and highly sensitive for detection of biomolecules (DNA, protein, peptide) by acting as luminescent probes in biological system [4]. In order to make QD compatible and flexible, the hydrophobic surface of QD needs to be modified in order to have hydrophilic functional group. The same functional group can be used for conjugation with biomolecules in aqueous media [5].

In analysis of biological application involving QD, it serves as energy donor in fluorescent resonance energy transfer (FRET) system. FRET is defined as through-space dipolar coupling interaction which allows electronic energy to be transferred from donor to acceptor [6]. QD has a broad absorption range and can be excited far from excitation range of the acceptor molecule and the absorption spectrum of acceptor can be paired with narrow emission of QD. By using this system, a specific DNA target can be detected where FRET can be used as signal for detection of positive hybridization.

In this report, we applied a FRET-based method utilizing QD for detection of synthetic DNA of *Ganoderma boninense*. In our approach, the synthesized QD is chemically modified in order to bind with the DNA probe. Cy5-labelled reporter probe, which is complementary with half of the target DNA, is used as acceptor in the FRET system. The FRET signal that can be observed after hybridization with the target DNA can be a marker for the existence of *Ganoderma boninense*.

## 2. Experimental Section

### 2.1. Material

Chemicals including octadecene, trioctylphosphine, selenium powder, toluene and 3-mercaptopropionic acid (MPA, 99%) were purchased from Sigma-Aldrich. 1-ethyl-3-[3-dimethylaminopropyl] carbodiimide hydrochloride (EDC, ≥98%) and N-hydroxysuccinimide (NHS, ≥98.5%) were purchased from Fluka. Potassium hydroxide (KOH) was purchased from Systerm. Acetone and ethanol were purchased from HmBG. Methanol was purchased from J.T.Baker (Center Valley, PA, USA). 

All DNAs were purchased from First BASE Laboratories Sdn Bhd, Selangor (Selangor, Malaysia) and their sequences are as follows:

Probe DNA-amine modified single stranded DNA (ssDNA) (20-mer):
  5′-/NH2C12/TGG GTT GTA GCT GGC CTT CC-3′
Complementary target DNA (35-mer):
  5′-GCT AGT CAA GGT AAC GGA AGG CCA GCT ACA ACC CA-3′
Non-complementary DNA (35-mer):
  5′-GTA AGG TGC TTG AAT TCG TTA GGC TTG GTT TCG AT-3′
Reporter probe (15-mer):
  5′-GTT ACC TTG ACT AGC/Cy5/-3′

All oligonucleotides (100 µM) were diluted using TE buffer (10 mM Tris-HCl, 1 mM EDTA, pH 8.0) before used as stock solutions and stored in frozen condition. The other solutions prepared were phosphate buffer saline (PBS) (137 mM NaCl, 10 mM phosphate, 2.7 mM KCl) (pH 7.4), sodium bicarbonate buffer (NaHCO_3_) (50 mM, pH 9.0) and phosphate buffer (20 mM, pH 6.0).

### 2.2. Method

#### 2.2.1. Preparation of CdSe QD

Thirty mg of Se, 5 mL octadecene and 0.4 mL trioctylphosphine were added together in a 10 mL round bottom flask. The solution was stirred and warmed as necessary to completely dissolve Se. Thirteen mg of CdO was added into a 25 mL round bottom flask clamped in a heating mantle. 0.6 mL oleic acid and 10 mL octadecene were then added to the same flask. The flask was swirled to mix the liquids together. The cadmium mixture solution was then heated to 225 °C. A clean and dry pipette was used to quickly transfer 1 mL of the room temperature selenium mixture solution to the cadmium mixture solution. 

#### 2.2.2. Preparation of Water Soluble CdSe QD

Water soluble CdSe QD was prepared by using ligand exchange reaction method which 500 µL of CdSe QD in toluene was reacted with 100 µL of MPA. The mixture was covered with aluminium foil and stored in the fridge for overnight. After this incubation time, 1 mL of 1.0 M KOH was transferred to the mixture and two phase layers were formed and the upper layer was discarded. Then, 1 mL of toluene was added to the remaining phase. The upper layer of the resulting emulsion was also discarded. Next, precipitation process was carried out to remove the excess of MPA by adding 1 mL of acetone to the solution for three repeated cycles. Precipitate obtained was then dissolved with 1 mL of phosphate buffer solution (PBS) and stored as stock solution in fridge for further usage.

#### 2.2.3. Attachment of CdSe QD with ssDNA (CdSe QD-ssDNA Conjugate)

The mixture of 200 µL 1:1 (v/v) EtOH/H_2_O was used to dissolved 30 mg of EDC and 15 mg of NHS which was then transferred into 200 µL of phosphate buffer (20 mM, pH 6.0). The mixture was then added with 200 µL of 1:1 (v/v) CdSe QD/H_2_O mixture. The solution was sonicated in a soniclean bath for 1 min before allowed to stand at room temperature for 1 h. The solution was then centrifuged at 4,000 rpm for 20 min and the clear supernatant was removed. The pellet was carefully washed with water (2 × 200 µL). Then, 175 µL of 1:1 (v/v) MeOH/H_2_O mixture was added to pellet followed by 25 µL of C6 amine modified DNA (440 µM) and 100 µL of NaHCO_3_ buffer (50 mM, pH 9.0). The mixture solution was sonicated for 2 min to break up the pellet and the solution was stored at 4 °C for overnight. After this reaction time, the sample was centrifuged at 4,000 rpm for 20 min and the clear supernatant was carefully separated from the pellet. The Pellet was washed with 200 µL of 1:1 (v/v) MeOH/H_2_O before 0.5 mL of deionized water was added and sonicated for 5 min to obtain a clear stock solution. It was stored in the dark at 4 °C until further usage.

#### 2.2.4. Hybridization

Hybridization solution of 1,500 µL which contain calculated amount of CdSe QD-ssDNA conjugate, reporter probe, target DNA and PBS buffer was mixed together in an eppendorf tube and shook thoroughly in order to obtain homogenous solution. The hybridization was carried out for 2 h at room temperature before fluorescence emission were taken. 

#### 2.2.5. Fluorescence Emission

Sample was transferred to a quartz cuvette with a micropipette. Fluorescence measurement for all samples was carried out by using spectrofluorophotometer (Shimadzu RF-5301PC, Kyoto, Japan). A fixed excitation wavelength at 488 nm with scan rate of 2 nm/s, bandwidth of 4 nm and slidwidth of 10 nm were used to obtain emission spectra (500–800 nm range) of all samples. 

#### 2.2.6. Transmission Electron Microscopy (TEM)

The samples particle size was measured using a HITACHI H-7100 TEM instrument. Samples of water soluble CdSe QD and CdSe QD-ssDNA conjugate were dropped on the copper grid separately for drying and spreading process. Then, the samples were measured with the magnification of 200,000× at scale of 100 nm. 

## 3. Results and Discussion

### 3.1. Water Soluble CdSe QD

Water soluble CdSe QD was reacted with MPA for overnight to make sure the hydrophobic ligands, hexadecylamine (HDA) and trioctylphosphine (TOPO) were exchanged with carboxylic (COOH) functional groups of MPA using ligand exchange reaction method. In this reaction, the hydrophobic ligands around surface of CdSe QD were replaced by hydrophilic ligands which contain thiol functional groups that attached to the surface of QD leading the COOH groups to be extended away from the QD. The prepared water soluble CdSe QD is stable up to one week. 

Ligand exchange reaction method used in this study was able to replace TOPO and HDA efficiently with thiols from MPA. The presence of carboxylic groups is important to enhance the solubility of QD in water. In the process of ligand exchange reaction to occur, the new ligands should have an affinity as strong as possible enough towards QD in order to quickly and effectively replace the original surfactant molecules. In this study, thiol groups bind strongly to the surface of CdSe QD replacing the weaker bond of TOPO and HDA hydrophobic surfactant. In principle, when MPA reacted with CdSe QD, its pH value decreases to a certain value at which TOPO and HDA were protonated and detached from the CdSe QD [7]. Strong affinity of the thiolated groups will enhance the binding capability of the hydrophilic ligands to the surface of CdSe QD. 

Normally, nanoparticle QD was modified for bioconjugation and to improve the optical properties. Since the organic ligand is not compatible in aqueous condition, the introduction of COOH groups enhance the compatibility of QD to covalently react with DNA in aqueous medium and increase its solubility in water. Besides, the coating protects QD from photo-initiated surface degradation, which is directly related to the fading of fluorescence intensity and toxicity.

### 3.2. Activation of COOH Functional Group on CdSe QD with EDC and NHS

Water soluble CdSe QD that contained COOH groups as bifunctional ligands was mixed with activating agents, EDC and NHS for formation of QD-ssDNA conjugate via amide-linkages. Normally, NHS has been used to prepare amine-reactive esters of COOH groups for chemical labeling, crosslinking and solid-phase immobilization applications. In this reaction, EDC was reacted with COOH groups of CdSe QD forming an unstable amine-reactive *O*-acylisourea intermediate which is also susceptible to hydrolysis, making it unstable and short-lived in aqueous solution. This intermediate was stabilized by the addition of NHS forming a semi-stable amine-reactive NHS ester. The COOH groups of resulting intermediate was then reacted with NH_2_ group of amine terminated ssDNA probe to form a stable amide bond and allowing conjugation of CdSe QD with ssDNA.

### 3.3. Sensing Characterization Based on Fluorescence Emission

**Figure 1:**
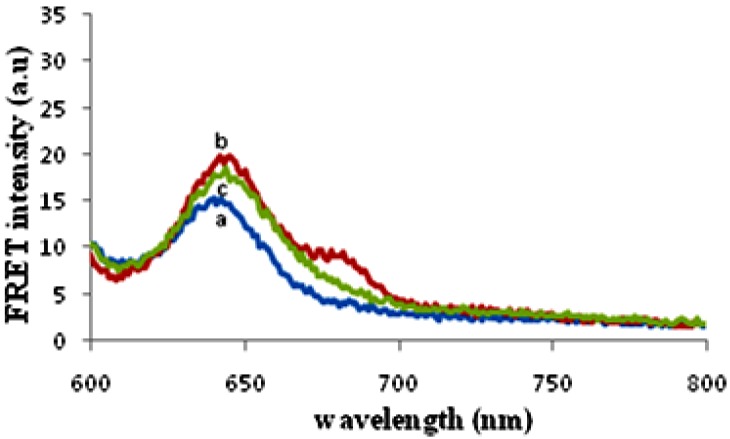
Fluorescence spectra of (**a**) CdSe quantum dot (QD)-ssDNA, (**b**) CdSe QD-ssDNA hybridized with complementary DNA and (**c**) CdSe QD-ssDNA hybridized with non-complementary DNA; Excitation was fixed at 488 nm; scan rate: 2 nm/s; slidwidth: 10 nm.

The changes of the fluorescence signals for CdSe QD-ssDNA conjugate and hybrid system contained target DNAs (complementary and non-complementary DNA) were used to characterize the sensing capability of the developed detection system. Figure 1 shows fluorescence spectra of CdSe QD-ssDNA and CdSe QD-dsDNA hybridized with complementary and non-complementary target DNA. The emission peak at 640 nm, which corresponds to the emission of QD, was obtained for all spectra. Only hybridization with complementary target DNA shows emission peak at 680 nm confirming the occurrence of hybridization. 

**Figure 2:**
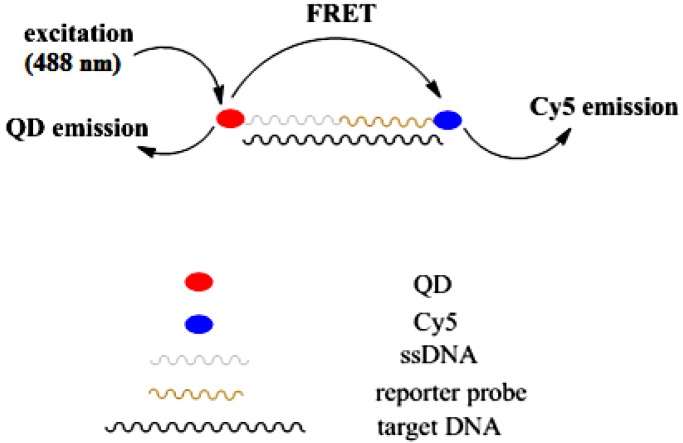
Fluorescence resonance energy transfer between donor QD and acceptor Cy5.

Hybridization solution contains two probes; reporter probe labeled with Cy5 and ssDNA conjugated with QD. Upon hybridization with target DNA, the reporter probe and CdSe QD-ssDNA will bind with the target forming sandwich hybrid [8]. Hybridization occurs in this system since the sequence of the ssDNA and reporter probe completely match with the sequence of target DNA (ssDNA match one half of the target sequence while reporter probe match the other half of the target). The resulting interaction of the hybrid brings the acceptor, Cy5 and donor QD into close proximity. When excited at 488 nm, QDs emit emission at 640 nm and lead the fluorescence emission from the acceptor Cy5 by means of FRET illumination, as shown in Figure 2. 

Therefore, the detection of fluorescence emission from acceptor Cy5 indicates the presence of target DNA and hybridization process that occurred in the solution. FRET emission was observed when hybridization was carried out with complementary target DNA but no FRET emission was observed when non-complementary target DNA was used for hybridization. This proved that the detection system is able to differentiate between complementary and non-complementary target DNA. In this case, non-complementary target DNA did not hybridize with ssDNA and reporter probe and QD is not in position for FRET emission.

### 3.4. Factors Affecting the Hybridization Signal Intensity

**Figure A1:**
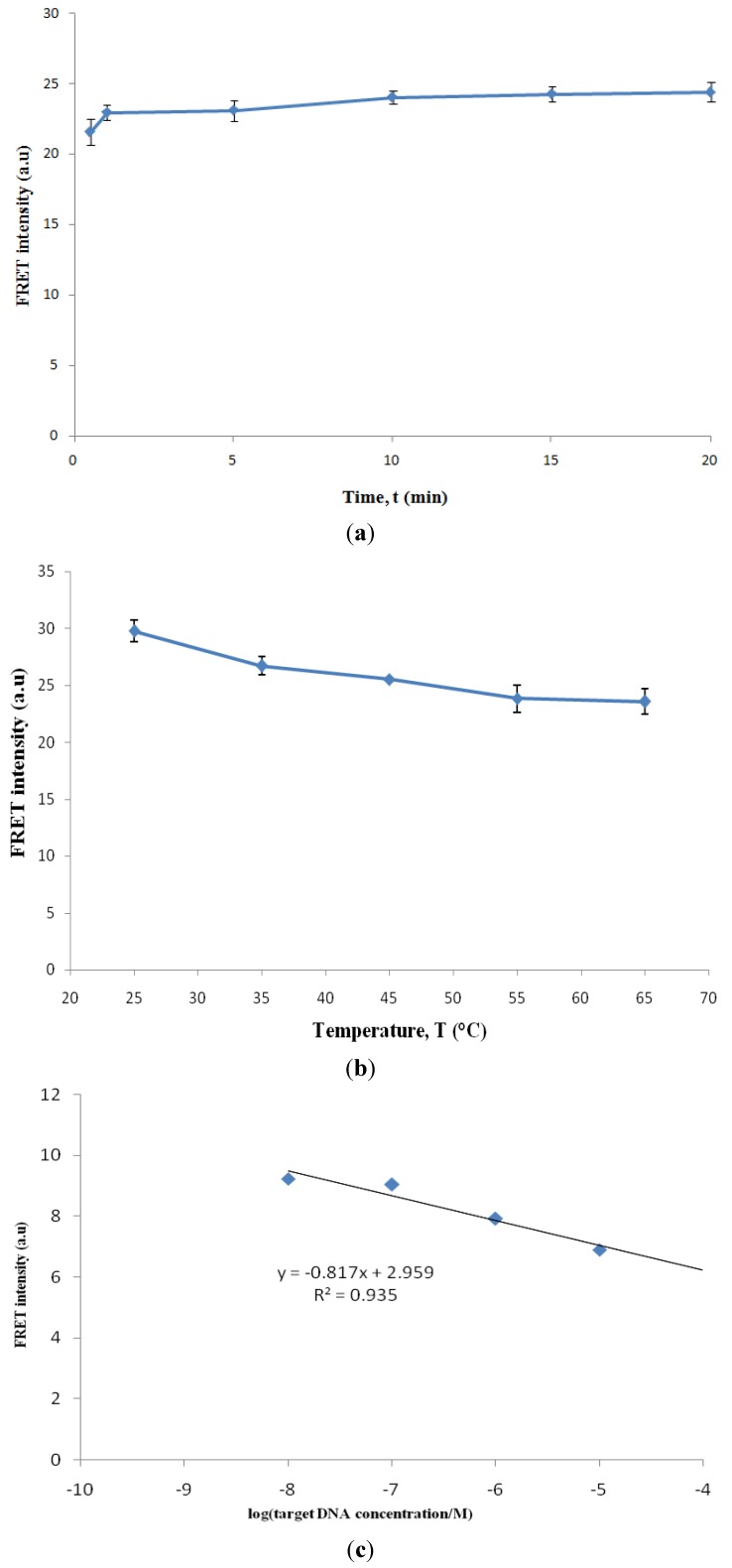
Factors affecting the hybridization signal intensity; effect of (**A**) hybridization time (**B**) hybridization temperature and (**C**) target DNA concentration on FRET emission. Excitation was fixed at 488 nm; scan rate: 2 nm/s; slidwidth: 10 nm.

Figure A1 (in Appendix) presented the factors affecting the hybridization signal intensity. The FRET intensity increases as the hybridization time increases ranging from 0.5 to 20 min. As can be seen, hybridization time of 10 min is enough to give significant FRET intensity for sensing purposes. Another important parameter is effect of temperature. Hybridization temperature is always discussed relative to the melting temperature of the given oligonucleotide. In this study, the effect of temperature of hybridization was varied from 25 to 65 °C. According to Figure A1(B), the intensity of FRET decreases gradually as the temperature increases. The highest FRET intensity was obtained when hybridization was performed at 25 °C. The hybridization of the DNAs was conducted along with QD that has been conjugated with ssDNA at optimized temperature. High temperature will affect FRET intensity since QD will lose its photoluminescence (PL) properties. Fluorescence decreases as temperature increases. This is due to the increase of molecular motion with increasing temperature, which results in more molecular collisions and subsequent loss of energy [9]. The sensitivity of developed DNA biosensor was studied using different concentration of target DNA. A plot of FRET intensity *versus* log concentration of target DNA in Figure A1(B) shows that FRET intensities decrease with respect to the logarithm value of the concentration of target DNA. The regression correlation coefficient (R^2^) is 0.935 and calculated limit of detection (LOD) for the developed system is 3.55 × 10^−9^ M. The decrease of FRET indicates the increase in negative charge density due to the formation of a DNA duplex which increases the repulsive electrostatic forces between QD and DNA resulting in larger distances and lower efficiency [10]. Besides, a high concentration of analyte will cause difficulty for the light to pass through the sample to instigate excitation. Thus, high concentration of analyte will have low fluorescence, which is known as concentration quenching [11]. 

### 3.5. TEM Characterization

**Figure 3:**
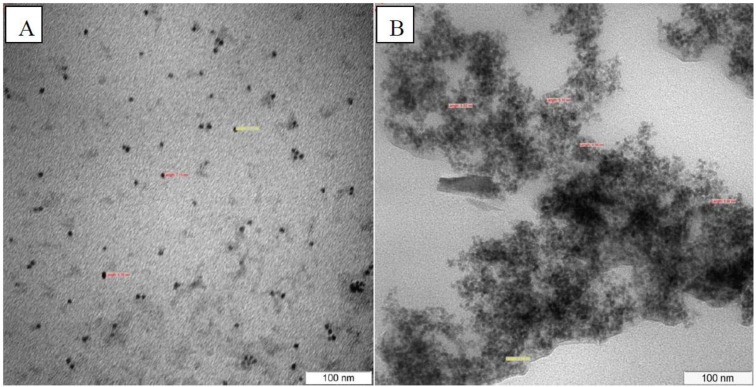
TEM image of (**A**) water soluble CdSe QD and (**B**) CdSe QD-ssDNA conjugate with magnification of 200,000×.

The particle size and morphology of water soluble CdSe QD and CdSe QD-ssDNA conjugate were analyzed using TEM. Figure 3 shows TEM image for both samples in 100 nm scale. It can be viewed that the size of the particles were in the range of 2 to 10 nm. These results also proved that the modification and conjugation of QD did not affect its particle size. It can been seen that in Figure 3(A), a single particle of QD is observed while in Figure 3(B), agglomeration is observed possibly due to conjugation with ssDNA. 

## 4. Conclusions

We have synthesized CdSe QD and applied the synthesized QD in development of DNA biosensor for synthetic DNA of *Ganoderma boninense*. Modification of QD retains the nano-sized of QD and compatible for bioconjugation with DNA. FRET involving QD was able to confirm the hybridization process which allows the detection of target DNA of *Ganoderma boninense* at high sensitivity. The developed DNA biosensor system has a potential for rapid, simple and sensitive application in agricultural and biological analysis. 

## Appendix

### Calculation of Limit of Detection (LOD)

Formula:

LOD = 3.3 (s/S) [12]
  s = Standard deviation of response (y-intercept of the regression line)
  S = Slope of calibration curve
  y = −0.817x + 2.959
  s = 2.092, S = −0.817
LOD = 3.3 (2.092/−0.817)
  = 8.45
  = anti log (−8.45)
  = 3.55 × 10^−9^ M
